# Potential for a web-based management information system to improve malaria control: An exploratory study in the Lahat District, South Sumatra Province, Indonesia

**DOI:** 10.1371/journal.pone.0229838

**Published:** 2020-06-09

**Authors:** Hamzah Hasyim, Firdaus Firdaus, Artha Prabawa, Pat Dale, Harapan Harapan, David A. Groneberg, Ulrich Kuch, Ruth Müller

**Affiliations:** 1 Institute for Occupational Medicine, Social Medicine and Environmental Medicine, Faculty of Medicine, Goethe University, Frankfurt am Main, Germany; 2 Faculty of Public Health, Sriwijaya University, Indralaya, South Sumatra Province, Indonesia; 3 Intelligence System Research Group, Faculty of Computer Science, Sriwijaya University, Indralaya, South Sumatra Province, Indonesia; 4 Department of Biostatistics and Population, Faculty of Public Health, Universitas Indonesia, Depok, Indonesia; 5 Environmental Futures Research Institute (EFRI), School of Environment & Science, Griffith University, Nathan, Queensland, Australia; 6 Medical Research Unit, School of Medicine, Universitas Syiah Kuala, Banda Aceh, Indonesia; 7 Unit of Entomology, Institute of Tropical Medicine, Antwerp, Belgium; Instituto Rene Rachou, BRAZIL

## Abstract

**Background:**

A web-based malaria reporting information system (MRIS) has the potential to improve malaria reporting and management. The aim of this study was to evaluate the existing manual paper-based MRIS and to provide a way to overcome the obstacles by developing a web-based MRIS in Indonesia.

**Methods:**

An exploratory study was conducted in 2012 in Lahat District, South Sumatra Province of Indonesia. We evaluated the current reporting system and identified the potential benefits of using a web-based MRIS by in-depth interviews on selected key informants. Feasibility study was then conducted to develop a prototype system. A web-based MRIS was developed, integrated and synchronized, with suitability ranging from Primary Healthcare Centres (PHCs) to the Lahat District Health Office.

**Results:**

The paper-based reporting system was sub-optimal due to a lack of transportation, communication, and human capacity. We developed a web-based MRIS to replace the current one. Although the web-based system has the potential to improve the malaria reporting information system, there were some barriers to its implementation, including lack of skilled operators, computer availability and lack of internet access. Recommended ways to overcome the obstacles are by training operators, making the application in an offline mode and able to be operated by mobile phone text messaging for malaria reporting.

**Conclusion:**

The web-based MRIS has the potential to be implemented as an enhanced malaria reporting information system and investment in the system to support timely management responses is essential for malaria elimination. The developed application can be cloned to other areas that have similar characteristics and MRIS with a built-in web base to aid its application in the 5G future.

## Introduction

Malaria is a public health problem in tropical and sub-tropical countries which associated with high morbidity and mortality, particularly in vulnerable groups [1, 2]. In 2017, it was estimated there were 219 million malaria cases globally, most of the cases occurred in Africa (200 million or 92%), followed by South-East Asia and the East Mediterranean region [3]. In Indonesia, the national government aims to eliminate malaria from the country by 2030 [4]. However, malaria is still a major public health problem in the country including in the Lahat District of South Sumatra Province. In 2012, the Annual Parasite Incidence (API) of malaria in Lahat District was 4.69 per 1,000 population [5]. The API is the most commonly used indicator for estimating the actual intensity of malaria transmission [6, 7]. Some determinants of malaria in the Lahat District have been identified including the proximity of breeding places of *Anopheles* mosquitoes to human settlement [8], as well as environmental factors that affect mosquitoes [9].

In the 1980s, the Ministry of Health (MoH) of Indonesia developed a paper-based integrated health centre reporting system, called *Sistem Pencatatan dan Pelaporan Tingkat Puskesmas* (SP2TP). However, after the implementation of the decentralization policy in 2004, the quality of and support for the health information system in each district and city decreased [10, 11]. This paper-based reporting system has not been well integrated into each health service unit such as in Primary Healthcare Centre (PHC) and District Health Office (DHC). Problems arise from the central, provincial and district/city governments in harmonizing policy implementation, including the synchronization, structuring and development of health information systems, and the commitment of regional governments to provide operational costs to implement essential health services [12]. Although online health information systems (OHIS) were established by the MoH in 2011, several factors have led to their failure and these are investigated in this paper. Because of delays in malaria reporting in endemic areas in the country, local transmission can increase as a result of late intervention in vector control and contact transmission surveys [13]. Therefore, it is essential to develop a rapid and accurate reporting system using a web-based malaria reporting information system (MRIS) by adopting open-source systems such as Joomla. Such a reporting system is consistent with the World Health Organization (WHO) guidelines for malaria elimination strongly advocating malaria surveillance and strengthening of the malaria information systems [14].

This study had two research questions: (a) What is the state of the current paper-based recording system for malaria? And (b) Is there a potential for improvement using a web-based system? Therefore, the primary objective of this study was to evaluate the existing manual paper-based MRIS including to assess the barriers in using it Lahat District of Indonesia. The secondary objective was to develop and implement an integrated web-based MRIS utilising the content management system (CMS) Joomla in order to enhance malaria reporting system.

## Methods

### Study site and study design

**Fig 1:**
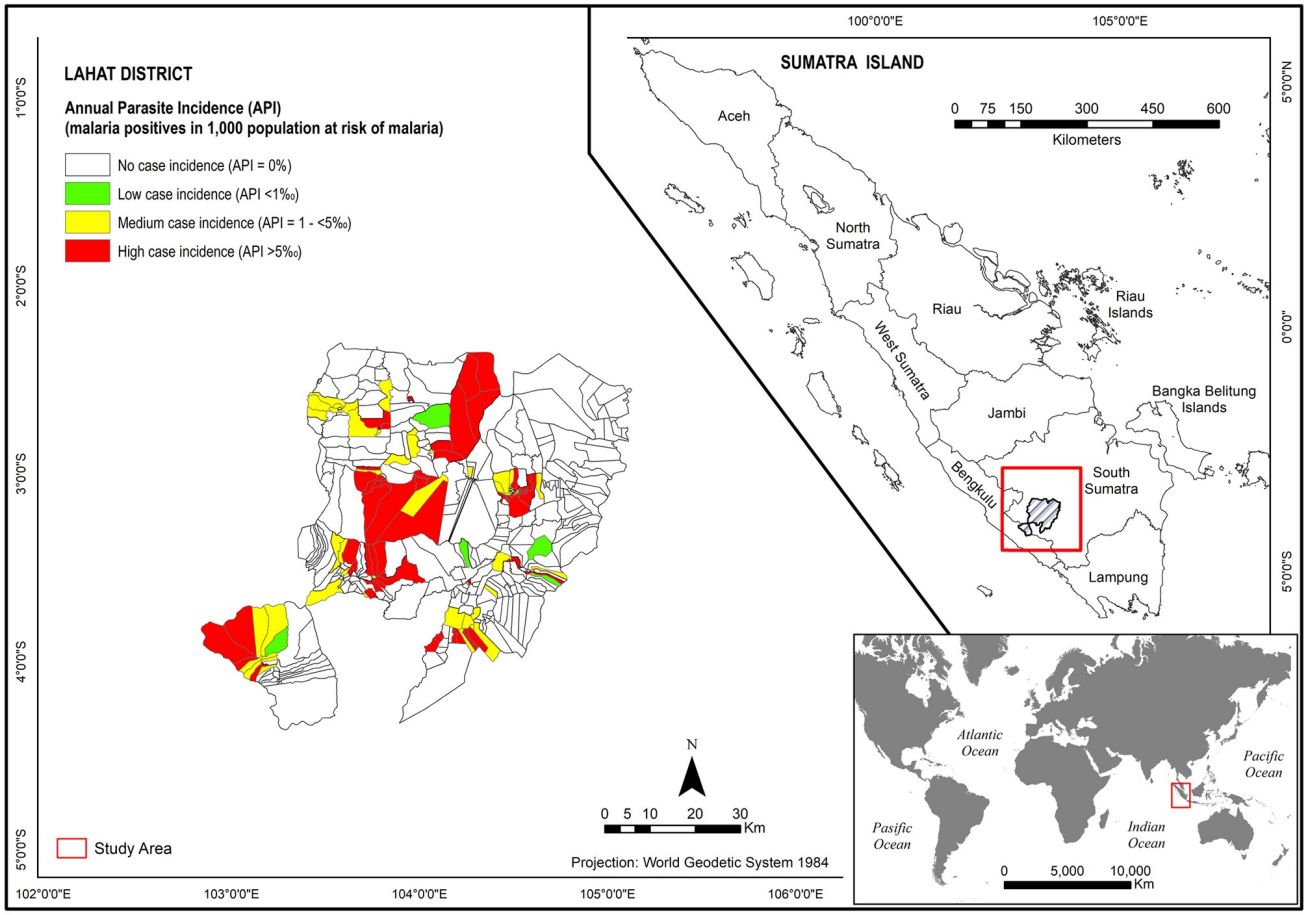
Map of the study areas (with permission from the indonesian the Geospatial Information Agency (BIG)).

Lahat District, an endemic malaria area in South Sumatra Province, is located between 1°46′ and 4°55′ of Southern Latitude and between 102°4′ and 104°41′ of Eastern Longitude and has a total surface area of 46,377.40 km^2^ (Fig 1). The Aeronautical Reconnaissance Coverage Geographic Information System (ArcGIS) software v10.3.1 was used for mapping, processing, analysing, and visualisation of the data set, and the World Geodetic System 1984 (WGS84) was used as the references coordinate system.

An exploratory study using in-depth interview approach was conducted in 2012 among the PHC directors and stakeholders who worked on malaria prevention and control program in the DHO of Lahat District. The interviews were conducted by investigators and aided by Public Health students. During the interview, documents related to the current paper-based MRIS were assessed including active and passive malaria surveillance documents, human resources, facilities, and related infrastructure document. In the next phase, a prototype of a web-based of MRIS was developed. In the final stage, prototype MRIS was tested by researcher who expertise in system information where it was integrated and synchronised ranging from the PHCs to the DHO.

### Key informant interviews

Interviews were conducted to obtain the perceptions of six key informants in Lahat District on using a paper-based MRIS, their perception of the need for a web-based MRIS, and their suggestions for MRIS development. Purposive sampling was employed to select the informants according to pre-determined categories, based on their knowledge and experience of using a MRIS. The key informants included the Heads of PHCs, the Coordinator of District disease prevention and control program, and District malaria officer who are directly engaged in the malaria program. The interviews were conducted by two researchers and helped by two undergraduate students of the Faculty of Public Health Sriwijaya University as enumerators who have been trained between June to July 2012. The training consisted of introducing data collection instruments, probing skills, recording responses, and transcription of records. Audio-tape and notes were recorded by all interviewers. The average time spent on each interview was approximately 30 minutes. The structured in-depth interviewing guidance is given in S1 Appendix.

### Analysis of qualitative data

Interview recordings were transcribed after the fieldwork. Themes were produced based on the following: (a) the MRIS which was used; (b) problems encountered in the paper-based MRIS activities and; (c) suggestions for the design and development of a web-based MRIS. The transcripts were then revalidated, and the transcribed notes were entered into the computer. The interview responses were further simplified by coding in order to organise, systematise the data and construct a picture of the topic [15]. In this study, the researcher used phrases, for example, "*accessibility and mobility*", "*technological affordability*", and "e*xpectation*" to represent the essence of the data segment. The computer transcript of every response was inspected for themes and compared with other interviewees to identify repetition words, relevant texts, and phrases. The variety of opinions and views of the interviewees collectively with their recognised related verbatim quotes were used to produce a narrative and outline of the findings.

### Development and testing of the web-based MRIS

Based on input from the informants in the review, we developed a prototype web-based MRIS at Sriwijaya University in Palembang utilising the content management system (CMS) Joomla. The information from in-depth interviews was synthesised and used as requirements of the basis for designing the system features. The final prototype was tested for its feasibility in the Laboratory of Health Informatics, Universitas Indonesia in Jakarta. The web-based MRIS was synchronised in one of sample Primary Healthcare Centres (PHCs) to the Lahat District Health Office.

**Fig 2:**
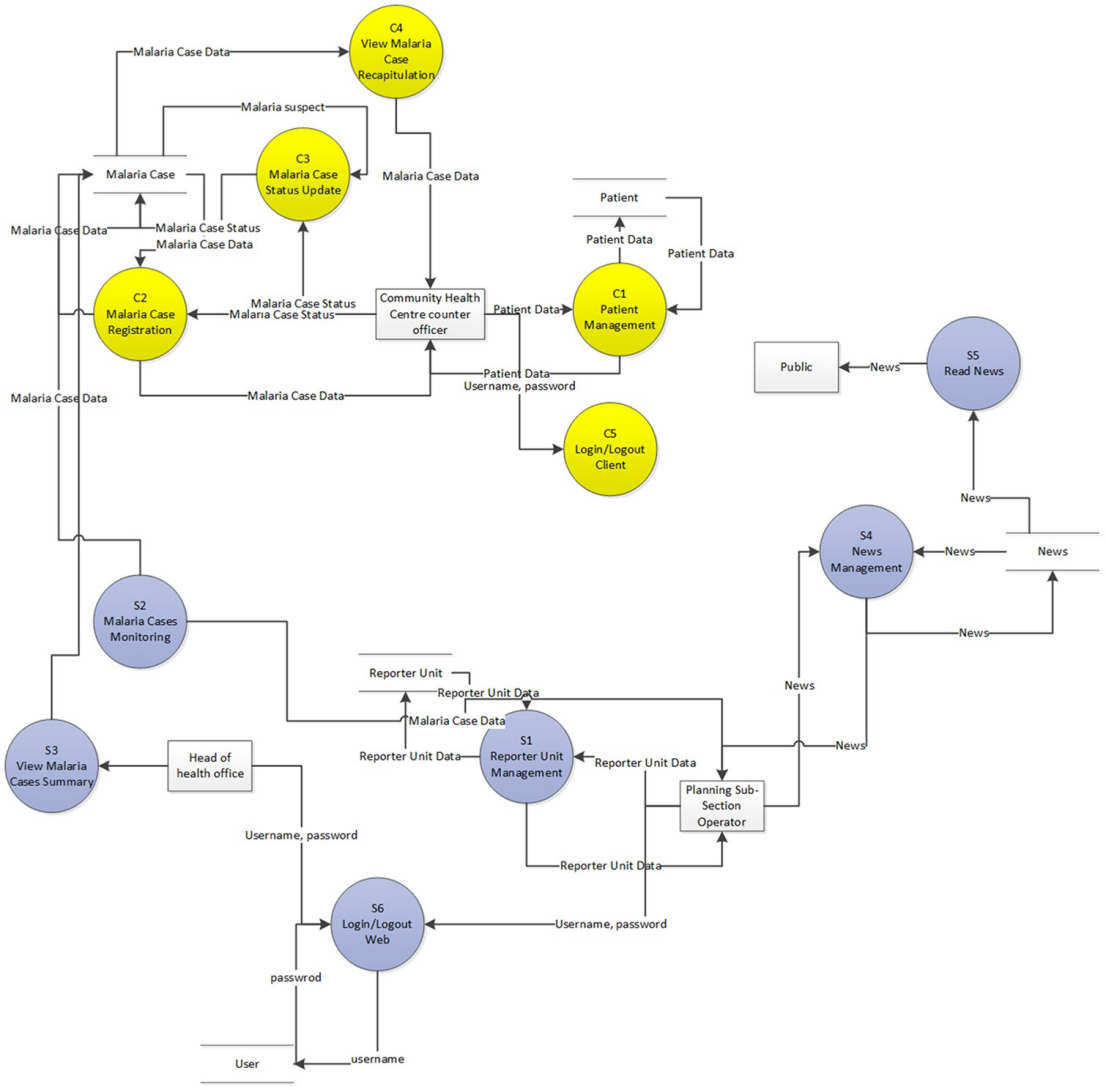
Data flow diagram (DFD) indicating the functionality of the information systems for web-based malaria reporting.

The MRIS was developed using a methodology Framework for the Application of Systems Techniques (FAST), a variation of the System Development Life Cycle (SDLC) [16–18]. FAST has an appropriate way of standardisation and has a stable process for understanding the system and for management planning. FAST consists of the following steps: (1) definition of the scope; (2) analysis of the problem; (3) analysis of needs; (4) the logic of design; (5) review of the decision; (6) physical design; (7) construction and testing; and (8) installation and delivery. The framework presents a general approach to a modular design that was the first stage of the SDLC. The data flow diagram of the developed web-based MRIS is presented in Fig 2. Detailed processes to operate the MRIS are provided in S2 Appendix.

#### Ethical considerations

The study was approved by the Research Institute of Sriwijaya University (168.a/UN9.3.1/PL/2012). Participation was voluntary in this research, and there was no financial incentive. The respondents provided written informed consent prior to participation.

## Results

### Evaluation of the existing paper-based MRIS

To evaluate the existing paper-based MRIS, in-depth interviews were conducted in six key informants in Lahat District. The results from this study were used as to develop new MRIS.

#### Characteristics of the key informants

We conducted a qualitative research study using in-depth interviews with a purposive sample of Heads of PHCs, the Coordinator of District disease prevention and control program, and District malaria officer. Using a non-probabilistic purposive sampling technique, we conducted interviews for six key informants. The type of key informant (e.g. policy-maker, epidemiologist) formed the unit of analysis. It served as the critical identifier allowing us to compare the perspectives of the kinds of informants. The characteristics of demographic variables from the key informants consist of five males and one female, who average on an age of 35 years old and five years duration of work that they have been in malaria elimination program at Lahat DHO. Besides that, the characteristics of participants in an education degree, one participant has a master education and others a bachelor degree in a public health program. Key informant interviews allowed us to solicit in-depth and candid opinions of a broad range of stakeholders effectively. Furthermore, qualitative research can identify rich narratives and lived experiences not captured in quantitative analysis and does make assumptions about MRIS literacy of respondents.

#### Main concerns related to existing paper-based MRIS

There were three main areas of concern raised by the informants: accessibility and mobility; technological affordability and expectation. The general overview from the research revealed weaknesses in the paper-based MRIS such as the difficulty of compiling and distributing paper-based reports due to transport issue. In in-depth interviews, the informants revealed their perceptions and experiences when using the paper-based MRIS. Their statements reflect complexity and delay in reporting malaria cases to DHO. Labour-intensive manual reporting impedes the accuracy of data reception at the district level.

Limitations of access to information challenges the reporting flow. Human capacity is a constraint, especially in computer operation. In every PHC, the lack of a skilled official is a real obstacle.

In summary, problems and difficulties encountered identify the potential benefit of and an urgent need for a web-based online open-source of MRIS. The main problems are: (a) difficulty in mobility and accessibility; (b) technology affordability; and (c) expectation. For all these issues there is a need for an urgent solution. The issues are described below using the direct reports of respondents in the three key areas.

*Accessibility and mobility*. In manual reporting, the speed of delivering data from the PHC to the DHO can determine the policy output at the provincial level but is impeded by the lack of transport facilities. In the Pagar Jati PHC, for instance, the reporting activities were run by the Head of the Administrative Office. Data reporting was sent out before the 5^th^ of every month. However, the subsequent information flow was also affected. The reporting format for malaria disease was separate from the template format provided by the Lahat DHO. Some of them were around its link-up with the PHC assessment and report of the SP2TP. The reporting process was completed manually for 2–3 days, this was subsequently recapitulated by the Diseases Prevention and Control (P2P). However, insufficient transport contributed to the inadequacies of and delays in the system:

*The shortcomings of the manual reporting system to Lahat DHO from Pagar Jati PHC is accessibility like (means of transportation that only operate once a day)*. *The distance between* Pagar Jati *PHC to DHO is 45 km with 1*.*5-hour trip*, *and the car only out once a day at 7–12*.

*(Head of Pagar Jati PHC)*.

Transportation facilities that were unable to extend to the Lahat District health office from the PHC office also caused delays:

*For surveillance activities*, *it has been done routinely and conducted by P2P officers*. *In addition to the data collecting*, *P2P officers perform data processing before it is expedited to* Lahat District *health office*. *Reporting of malaria data at Tanjung Sakti PHC usually refers to the form provided by Lahat* District *health office and finished before the 5th of each month*. *Constraints the staff overcome during the reporting is the risk of delay in delivery of reports*.

*(Head of Tanjung Sakti PHC)*.

The monitoring process in the Fajar Bulan Subdistrict also experienced delays due to the long distance. The lack of transportation facilities also curtailed the delivery of final reports:

*Given a wide working area*, *it may take a long time in the surveillance process*. *Also*, *there is a delay in the process of collecting reports because the officer must travel for 2*.*5 hours or 67 kilometres to Lahat DHO*.

The findings confirm that the lack of both public transportation and cars operated by PHC staff have contributed significantly to the frequent delays in the final report delivery to Lahat District health office. As well, hard-to-navigate terrain presents further obstacles to access. Insufficient funding for operating transportation is construed to add to the current problems.

*Technological affordability*. Technology applications in each PHC have encountered fundamental challenges that require an immediate solution. This obstacle originates from the unavailability of mobile phone and internet signals:

*We only have one laptop and rely on GPRS network with a personal modem and not 3G*. *There are often no networks*.

(Head of Pagar Sakti PHC)

In addition, the infrastructure that has long been in place is often disrupted. He continued their testimony:

*There is one tower but often not working*. *The intermittent disruption usually occurs for up to 3 days*.

Even though mobile phone signals can be good, difficult terrain limits the provision of infrastructure:

*Mobile phone receiving a signal in PHC*’*s are strong enough*, *but the cable network cannot enter our area*

*(Head of Tanjung Sakti PHC)*.

Computer operation is also constrained by human resources in every PHC limitations of access to information due to the lack of internet networks that transfer knowledge, challenge the reporting flow.

The Head of the Tanjung Sakti PHC shared him concerns:

*We have got three computers from the health service*, *but lack of human resources who can operate the computer*

*(Head of Tanjung Sakti PHC)*.

Manual reporting components that depend on data variables were also not reconciled in the field. The situation at the PHC of the Tanjung Sakti Subdistrict is:

*Laboratory equipment is incomplete*, *and there is no analyst*. *Finally*, *we employ a nurse as a to work in the lab*. *Chemicals are also lacking*, *and laboratory is less regularly used*

*(Head of Tanjung Sakti PHC)*.

In more detail, the Head of the Tanjung Sakti PHC iterated further that evidence on insufficient input variables within the reporting system:

*Clinical symptoms data*, *data from laboratory results are not comprehensively made available*. *Complications*, *treatment*, *environment (close to river water*, *housing conditions*, *home ventilation*. *We like to identify the origin of the population whether they came over to move in*, *attend schools*, *or come from other regions*. *The availability of the drug is not enough*. *The case reports*, *as well as clinical symptoms*, *are more critical actually*

*(Head of Tanjung Sakti PHC)*.

On the other hand, labour-intensive manual reporting also impedes the accuracy of data reception at the district level:

*Reporting delays are caused by manual systems maintained*, *and the amount of human resources to compile reports is still low*

*(Head of Tanjung Sakti PHC)*.

The lack of funds for procuring new computers is also a point to note before on-line reporting is applied in the field. They continued:

*If online reporting is applied*, *then we are constrained by the inadequate funding issue of buying a computer*.

Technological affordance is complex and multi-layered. As described in the narrative, some of the problems that need to be immediately resolved are the access to internet signals and mobile phone receivers, providing the number of computers according to requirements and providing well-trained staff at every PHC.

*Expectation*. The experience of the PHCs in the Lahat District depicts the limitations of malaria handling and monitoring, despite being handled with standard procedures, which have long been locally practised and run by health professionals at the district, village, and subdistrict levels. Institutional support in the form of technological innovation is yet to be implemented at the local level but is expected to be applied sooner rather than later to remove limitations in the reporting. Such a situation puts the PHC under pressure to utilise on-line reporting in the field:

*With this online reporting*, *we will be able to report to the health centre in Lahat District*

*(Head of Pagar Jati PHC)*.

On the other hand, the speed of handling is the highest expectation observed in the results of the online reporting design:

*Later on*, *if the reporting system is running fast*, *the problems we face will get a rapidly responsive solution as well*

*(Head of Tanjung Sakti PHC)*.

At a higher level, accurate policy-making is desirable and used to support the professionalism of the surveillance officers each month:

*If there is an extraordinary occurrence of malaria from surveillance reports*, *immediate evaluation and action can be undertaken*. *With online reporting system*, *the PHC can be involved in higher-level policy-making*

*(Head of Fajar Bulan PHC)*.

The implementation of on-line reporting cannot be instantaneous. It needs a transition period that allows for a smooth transformation from paper-based mechanisms to the internet-based system. The Head of the Fajar Bulan PHC echoed:

*The reporting system is not a problem because it does not require electricity in the process*. *It just needs to be modernised so that reporting is not complicated*

*(Head of the Fajar Bulan PHC)*.

This narrative indicates that the modernisation of reporting via the internet is indispensable, but also that the manual reporting must be condensed. A fatal case of malaria treatment at the local level is typical; such cases would be a “*red alert*” for the high-level institutions at the district level. This would prompt them to formulate prevention policy for the concerned area more evenly and quickly. The fact that the Tanjung Sakti Subdistrict had an outbreak is a priority case to be addressed:

*Moreover*, *online reporting played a significant role in Lahat DHO*. *PHC’s are supportive if there is an online reporting system because the information submitted to the health service will be faster*, *primarily when outbreaks are found out*. *Delays are fatal*, *because if at the* district *level late it will be difficult to handle at the provincial level*

(Head of Tanjung Sakti PHC)

This narrative reveals the urgent demand for implementing on-line reporting for the Tanjung Sakti Subdistrict to deal with outbreaks. Despite high demand of the PHC’s for creating and implementing an on-line reporting system, the accessibility of internet signals in the field still concerns policymakers at the PHC level in those subdistricts. The Head of the Fajar Bulan PHC welcomed the application of Joomla as an on-line reporting system for malaria eradication:

*Joomla*, *as part of the malaria eradication program*, *is excellent*, *but unfortunately*, *there is no internet connection those living in remote areas*. *So*, *if you want to employ IT staffs for it*, *the infrastructure also needs to be improved*

*(Head of Fajar Bulan PHC)*.

This technological innovation is an intrinsic product of knowledge development. The Head continued with optimism, *"This kind of program is quite well implemented as the science progresses"*.

#### Development and testing of web-based MRIS

Data analysis uses the System Development Life Cycle (SDLC) approach methodology, which is one of the methods in software development. System Design conducted in the Laboratory of Health Informatics, Universitas Indonesia in Jakarta. Components of the prototype feasibility test used in this study is shown in S1 Table

Web-based of MIRS has a database that can be accessed quickly, from the results of this access speed test depends on the capacity of the hardware that supports the system. The ability of the kind of MRIS to store data in real-time must be supported by input security features which must still be upgraded. Some of these advantages of web-based of MIRS developments strengthen recording and reporting capabilities at the district level.

## Discussion

Our study indicated that the paper-based MRIS is inefficient or ineffective because some reasons mainly related to accessibility and mobility, technological affordability, and expectation. We recommend that the manual paper-based model should be replaced with an electronic reporting system. The web-based reporting system using Joomla is one of feasible alternative to accelerate collecting and analysing malaria incidence data in the DHO. So, to bring this web-based system to the customer is requiring human resources management and training and enhancing network infrastructure, which refers to the composite software and hardware, including network resources and services.

The web-based reporting system using Joomla system has several benefits. A major advantage is that the form can be upgraded by including modules developed in many applications by other information and communications technology (ICT) system developers and uploaded on various open-access sites. The open-source programming language was first released in 2005 and has seen upgrades since then, but remains a free, open source system that can be used for a MRIS. It will remain up-to-date because it is an innovative ICT system, continuously being developed by communication practitioners and academics in various fields. The applications are resistant to computer viruses, which could otherwise significantly impair the system. The development of information systems using an open-source system also facilitates the development of an interdisciplinary model to maximize the scope of the application [19].

However, the implementation of the web-based reporting system using Joomla is limited due to lack of internet access and infrastructure and these must be improved and made reliable, with priority to remote areas. To realise the benefits of the internet, the government should forge a partnership with state telecommunication companies to build internet installations [20]. By the cooperation in building internet installation would address expectations of Head of PHCs for increased speed of data handling resulting from recent technological advances that would be provided by the on-line reporting system. More generally, making a web application or Short Message Service (SMS) gateway can supply feedback in the form of a decision based on the standards for the prevention and eradication of diseases [21]. A study in Papua New Guinea demonstrated that the use of mobile technologies and Geographical Information System (GIS) in capturing and reporting of national health information system (NHIS) data provides timely, high quality, geocoded, case-based malaria data, useful for malaria elimination [22]. Similarly, the data system encouraging malaria elimination involves: quick and comprehensive case reporting, integration of associated knowledge such as a health information system, automatic and skilled info analysis, and tailor-made outputs and comments that contribute to timely and targeted solutions [23].

At a higher level, accurate policy-making should support the professionalism of the surveillance officers each month. Training is a critical component of this. Access to training and support, and availability of hardware including computer and system receivers is critical [20,24]. An appropriate number of staff who are medically informed and technologically competent are urgently required to solve the problems identified in the research [25]. As an example, according to the problems reported in the present research, health offices need to identify the priorities for improving the skills of medical personnel using web-based MRIS, the distribution of stable internet connections, including the availability of standardised computers, the provision of transportation facilities, and obtaining sufficient budget arrangements in every PHC. Acceptability of the reporting process is crucial from PHC to DHO. All stakeholders’ needs should be identified with the role of each actor in the PHC’s including Head of PHC’s, the coordinator of district disease prevention and control program, and district malaria officers.

The involvement of each actor is essential to ensure flexibility [26], sustainability and innovation of web-based reporting [27].

Using the CMS Joomla system users can quickly obtain information about the surveillance and services existing across activities, particularly regarding the area-based management of malaria eradication. Thus, the malaria situation in an area can be determined through the collection of precise data to help determine countermeasures as soon as possible. This would create up-to-date, and accurate information which help provide efficient and effective decision-making. A reliable and useable surveillance system is essential for malaria elimination as demonstrated in the following two studies. Globally, the mapping of the distribution of malaria was able to capture at-risk population groups to control malaria transmission [28]. In Vietnam, the health information systems development was a critical component of disease control, crucial for disease risk assessment, formulation, and evaluation of priority of different interventions in the cost-effectiveness of malaria cases more than ten years ago [29]. Bhutan’s experience of integrating web-based and mobile technology to map data surveillance and generate real-time reports should be taken as the best example for speeding the country-level decision-making process and reducing malaria rates [30].

Finally, the elimination of malaria can be achieved not only with the key early and effective treatment, the prompt and accurate diagnosis of malaria, and rapid diagnostic tests (RDTs), but also by the strengthening of MRIS that is facilitated by training and accurate information gathering, including increased awareness and the utilisation of insecticide-treated mosquito nets [31–33].

## Conclusions

Our study indicates that the current paper-based MRIS in Indonesia is suboptimal because of the complexity and difficulties in handling reporting MRIS manually. This can be remedied by implementing the web-based MRIS. The implementation of a web-based reporting system using Joomla will potentially improve malaria reporting and management and it therefore could accelerate the progress of malaria elimination in Indonesia.

## Supporting information

S1 Appendix Detailed instrument of study for in-depth interview. (DOCX)

S2 Appendix Detailed processes on how to run the web-based MRIS. (DOCX)

S1 Table Components of the prototype feasibility test. (DOCX)
