# Supplementary material for: Potential for a web-based management information system to improve malaria control: An exploratory study in the Lahat District, South Sumatra Province, Indonesia
Source: PLoS One. 2020 Jun 9;15(6):e0229838. doi: 10.1371/journal.pone.0229838 (PMC7282623; doi:10.1371/journal.pone.0229838)
Supplement: S1 Appendix — (DOCX) [file pone.0229838.s001.docx]

# S1. In-Depth Interviewing Guidance

# In-Depth Interviewing Guidance

**Potential for a web-based management information system to improve malaria control using the content management system (CMS) Joomla.**

**The key informants are Director of PHC, the coordinator of district disease prevention and control program, and district malaria officers.**

- - 1. **Introduction**
- Describe the purpose of the discussion:

“We came here to get an explanation or elucidation from you concerning public health issues in this area, and the implementation of the malaria reporting information system (MRIS), as part of the malaria elimination program, particularly where you lead in these work areas. The information that we obtain is solely used for academic purposes and will be kept confidential (i.e not available to a wider audience including the public).

- - 1. **Description of the interview**

| Interview date | : |  |
| --- | --- | --- |
| Interviewer's name | : |  |

- - 1. **Characteristics of key informants**

|  | Name | : |  |
| --- | --- | --- | --- |
|  | Age / duration of work | : |  |
|  | Higher education | : |  |
|  | Occupation / position | : |  |
|  | Office address | : |  |

signature,

  (.............................................)

**Iv. Purpose**

General:

The primary purpose of this study is to assess barriers to use the current paper-based malaria reporting information system (MRIS). The secondary goal was to develop and implement an integrated web-based MRIS utilising the CMS Joomla system in the Lahat district of Indonesia.

 Specific:

- 1. Describing the current paper- based MRIS.
  2. Evaluating the problems faced by the paper-based MRIS.
  3. Recommending ways to overcome the problems by developing a web-based system consistent with the perspectives/ expectations of key stakeholders.
  4. Developing a web-based MRIS that can be used for input, process and output activities.
  5. Conducting a feasibility trial of the web-based of MRIS using the CMS Joomla system that integrates and synchronises information, ranging from the Primary Healthcare Centers (PHC) to the Lahat district health office.

**Iv. Question material**

1. **Describe the situation and the stem malaria reporting information that is currently used**
2. What was the annual parasite incidence (API) in the past year, in Lahat district health office?
3. Do you think the API has increased in this area? If yes, what do you think is the cause of the increased API
4. **Knowing the problems encountered in malaria reporting activities**
5. One policy and strategy for malaria elimination program is conducting a malaria reporting system. To what extent has these activities been carried out in your area?
6. What are the obstacles to the implementation of the reporting system?
7. What is the policy regarding an MRIS in Lahat district health office, what departments are involved in this activity, and what is the mechanism?
8. Are there special programs carried out for these activities that can be used between sections (for example disease prevention and control program and data processing)?
9. What kind of data has been collected, both in the PHC and Lahat district health office? How is the data collected?
10. What is the support of the local government in developing a MRIS as part of the malaria elimination program?
11. What is your role and responsibility in determining the policy for developing MRIS, as part of a malaria elimination program?

1. **Design / develop malaria reporting forms as needed.**
2. How has MRIS been implemented?
3. What is the form of the current MRIS, and what are the benefits and constraints?
4. In general, what is your view of the ongoing manual malaria reporting such as input activity, process and output in this region? Is it necessary improve it? What things need to be added or improved?
5. In general, what is your view of implementing a web-based MRIS which is integrated and synchronised, as part of a malaria elimination program, ranging from the PHC to the Lahat district health office?
6. Do you need to proceed it? What things need to be added or improved?
7. Has the web-based MRIS been implemented using the CMS Joomla as part of the malaria elimination program (Note: Interviewer explained about the web-based MRIS program previously)
8. What did you think when the web-based of MRIS was implemented? Is the program useful for malaria elimination in the Lahat district health office?

**Observation Documents**

1. Document of active and passive surveillance of malaria
2. The documents of human resources, facilities, and infrastructure related to the development of MRIS including PC facilities
3. Form of paper-based MRIS
