# Supplementary material for: Potential for a web-based management information system to improve malaria control: An exploratory study in the Lahat District, South Sumatra Province, Indonesia
Source: PLoS One. 2020 Jun 9;15(6):e0229838. doi: 10.1371/journal.pone.0229838 (PMC7282623; doi:10.1371/journal.pone.0229838)
Supplement: S2 Appendix — (DOCX) [file pone.0229838.s002.docx]

# Supporting information

# S2. Detailed processes on how to run the web-based MRIS

Malaria Reporting Information System (MRIS) is a software used for reporting malaria case in the Lahat district health office. The web-based MRIS is divided into two separate applications, namely the software web-based application used by the Lahat district health office and the software used for reporting units in the form of a desktop application that is connected to previous requests. The physical architecture for a web-based MRIS can be seen in Figure 1.


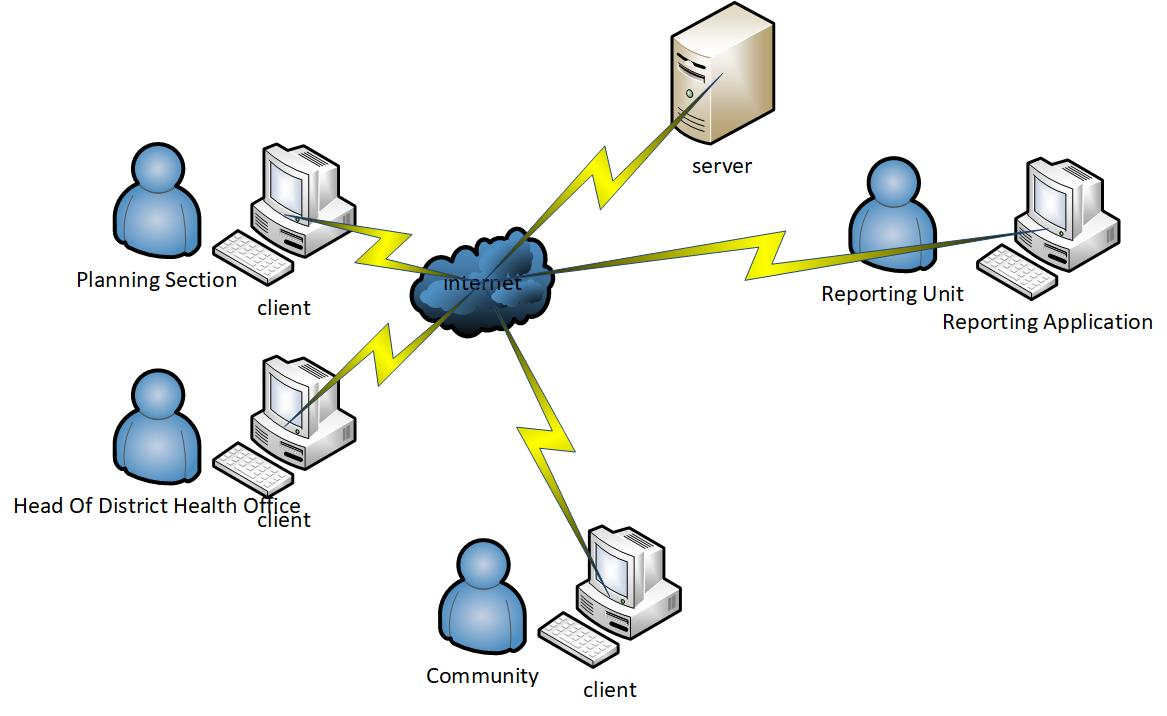


## Figure 1

Physical Architecture of the MRIS

The reporting of malaria cases is carried out by reporting units such as primary healthcare centres (PHC) in each sub-district, regional hospitals, private hospitals, health clinics, doctors' practices and other health care facilities, whereas the control function is carried out by the Lahat district office by looking at the recapitulation and producing graphs of malaria incidence throughout the Lahat District. In addition to the two functions above, there is also the function of delivering information on events and news to the broader community.

**Reporting Unit**

To enter the reporting unit of a web-based version of MRIS, users must first log in; for administrator type users, this is done by filling in in your username and password (other user types can also log in using the same procedure).


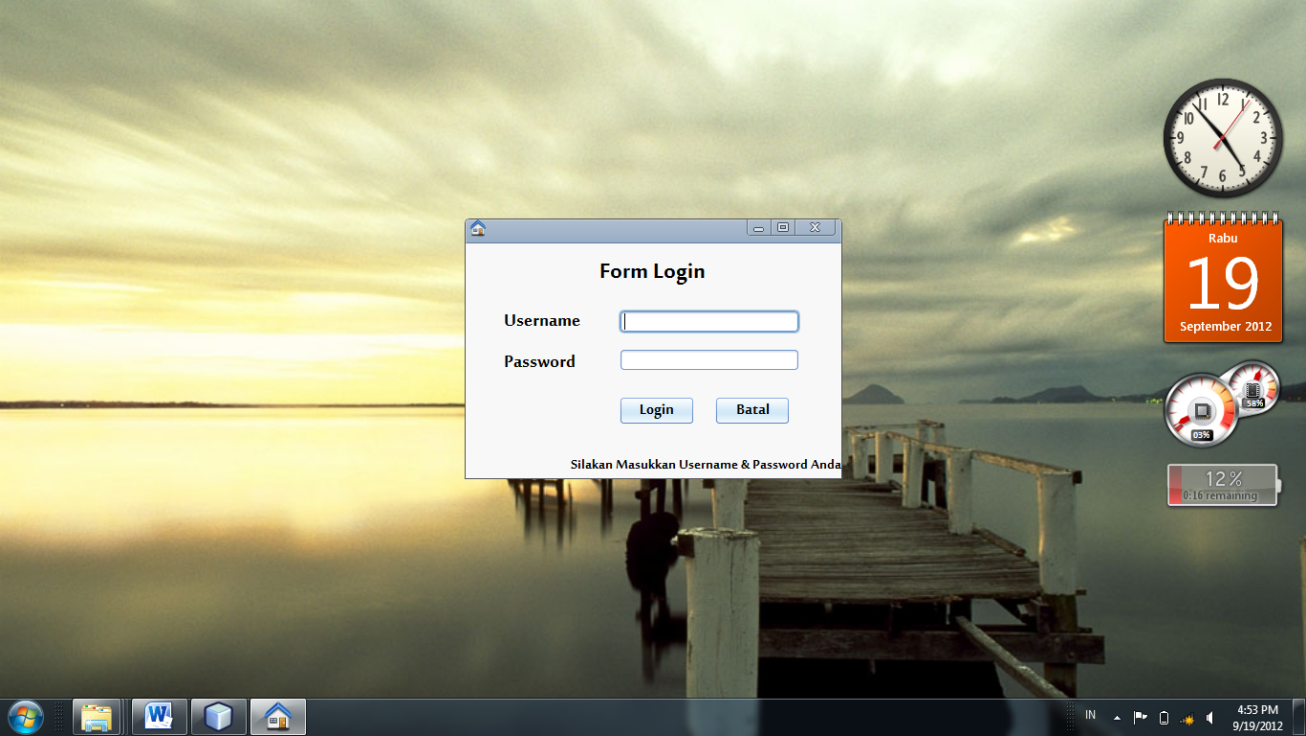


## Figure 2

After logging in successfully, the main menu of the application's home page will appear as follows:


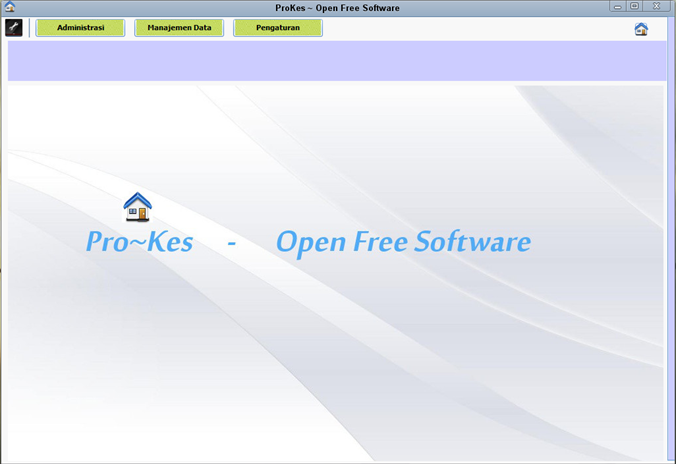


## Figure 3

The main menu display of the Pro~Kes (Health Information Systems) application is in the form of a ribbon, similar to those of the latest Microsoft Office software, the purpose of which is for ease of use and navigation.

There are three main menus in this application. Each main menu consists of several sub-menus that will appear on the ribbon panel if the main menu is selected.

1. **Administration Menu**

This menu consists of two sub-menus, namely:

1. Registration

The registration menu display is as follows:


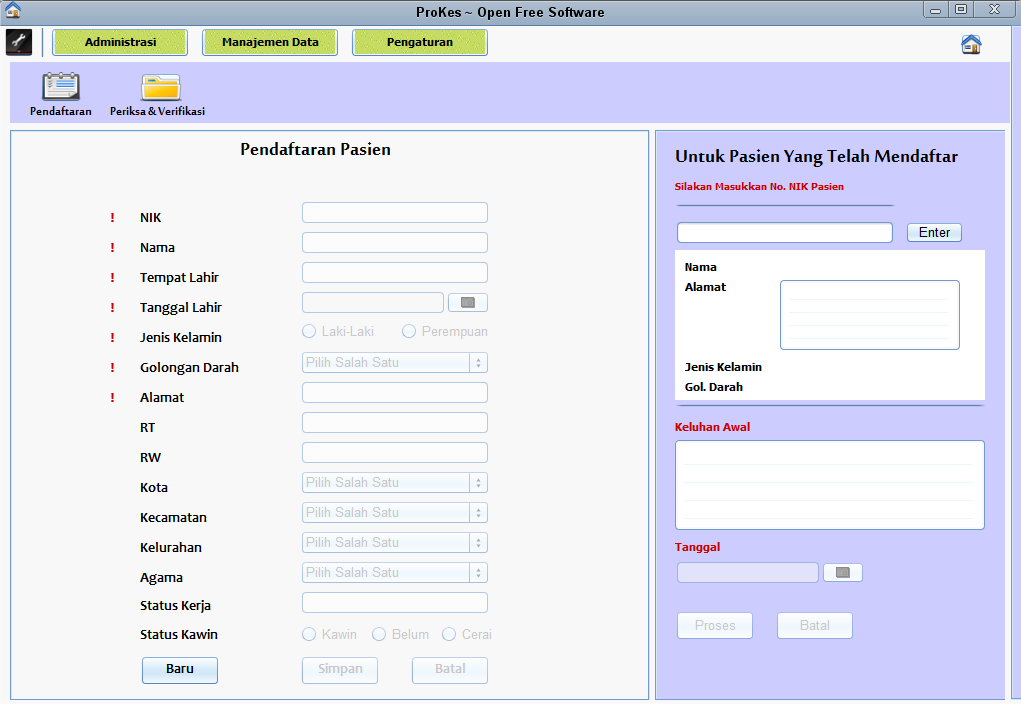


## Figure 4

Patients who first seek treatment, must first register with the hospital staff; the officer then fills in the data fields in the application according to the patient's ID card.

After registering, the officer can enter the patient's ID card number data, as stated on the ID card, in the appropriate column on the page, see Figure 5 then press enter. The application will automatically select and display the relevant patient data from the existing database.

1. Check and Verify

If the patient has registered, his /her name will automatically appear on the patient register, listed in this sub-menu.


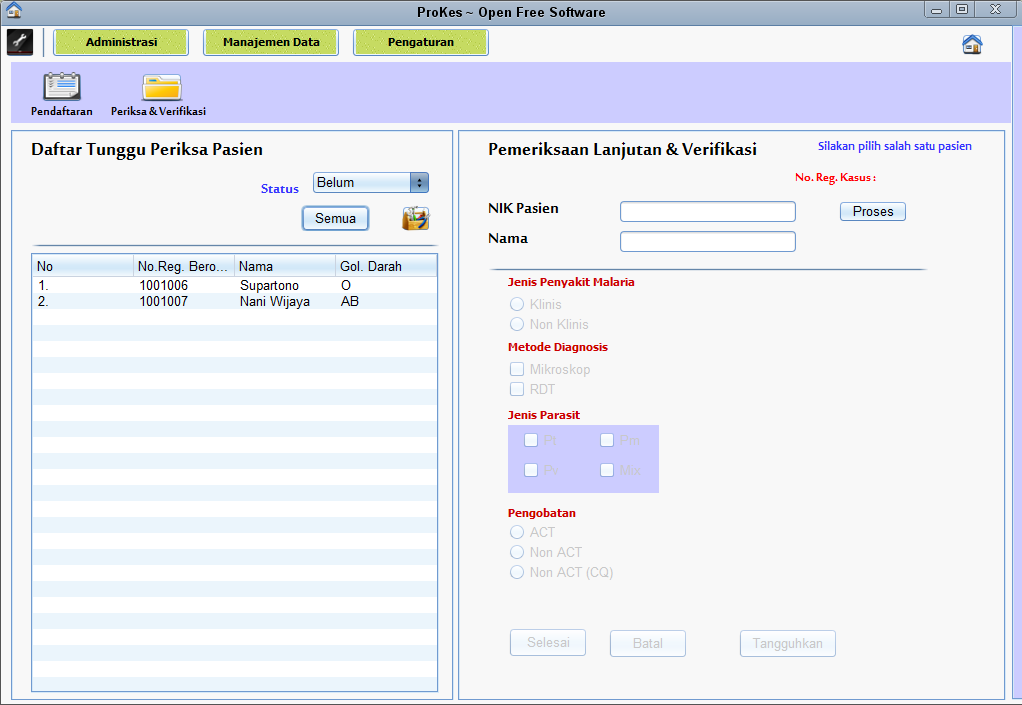


## Figure 5

Data shown here can be sorted/filtered according to requirements, the date or other patient data. To display other options or parameters, one can press the colourful equipment button and several alternative options will appear to sort the data.


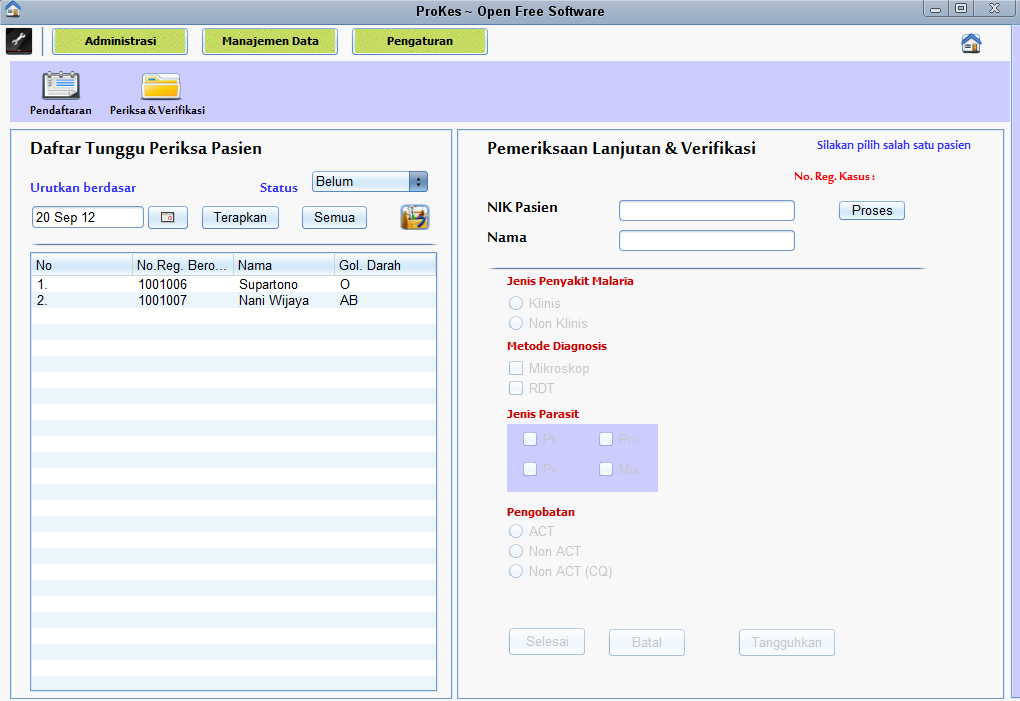


## Figure 6

Keeping in mind that this colourful tool button is also present in the other sub-menus of this application, it can be pressed to bring up some alternatives that may be needed.

To process a patient examination, select one of the patients by clicking on the name listed in the table. Now press the process button located on the right side of the application panel. Fill in the inspection data in the appropriate column, then press the complete or cancel button.

1. **Data Management Menu**

This menu consists of three sub-menus, namely:

1. Data Treatment


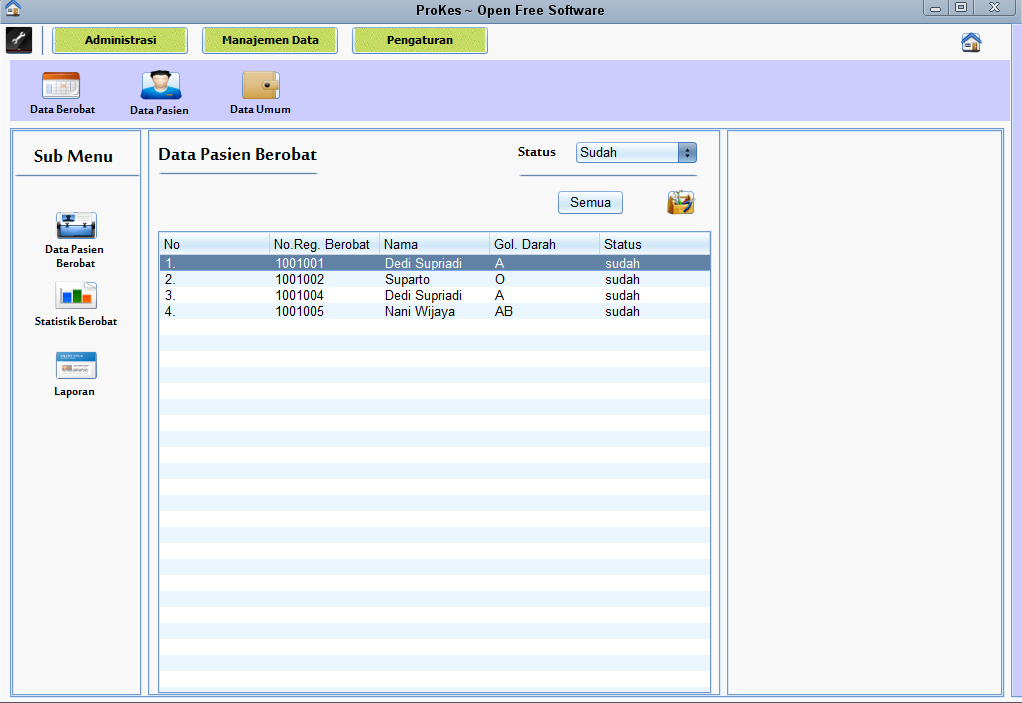


## Figure 7

The medical data sub-menu consists of three sub-menus, namely:

1. Patient data treatment

Displays data of patients seeking treatment at the relevant health agency unit.

1. Medical treatment statistics

Displays treatment statistics, either daily, monthly or according to the parameters chosen by the user.

1. Report

Displays data reports and also a preview which can then be printed or saved as a file in a particular format on the hard drive.

1. Patient Data


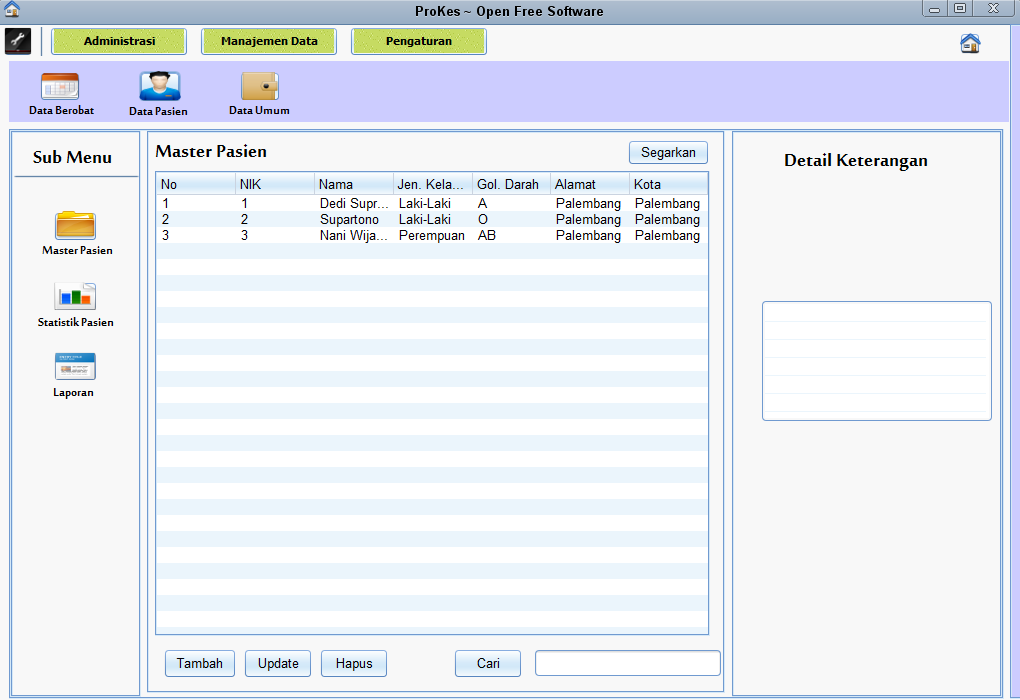


## Figure 8

The patient data sub-menu consists of three sub-menus, namely:

1. Patient master

The patient master displays the patient data that has been officially registered with the existing health unit.

1. Patient statistics

Displays statistics on the number of patients when requested

1. Report

Displays data reports and also a preview which can then be printed or saved in a file with a format on the hard drive.

1. General Data


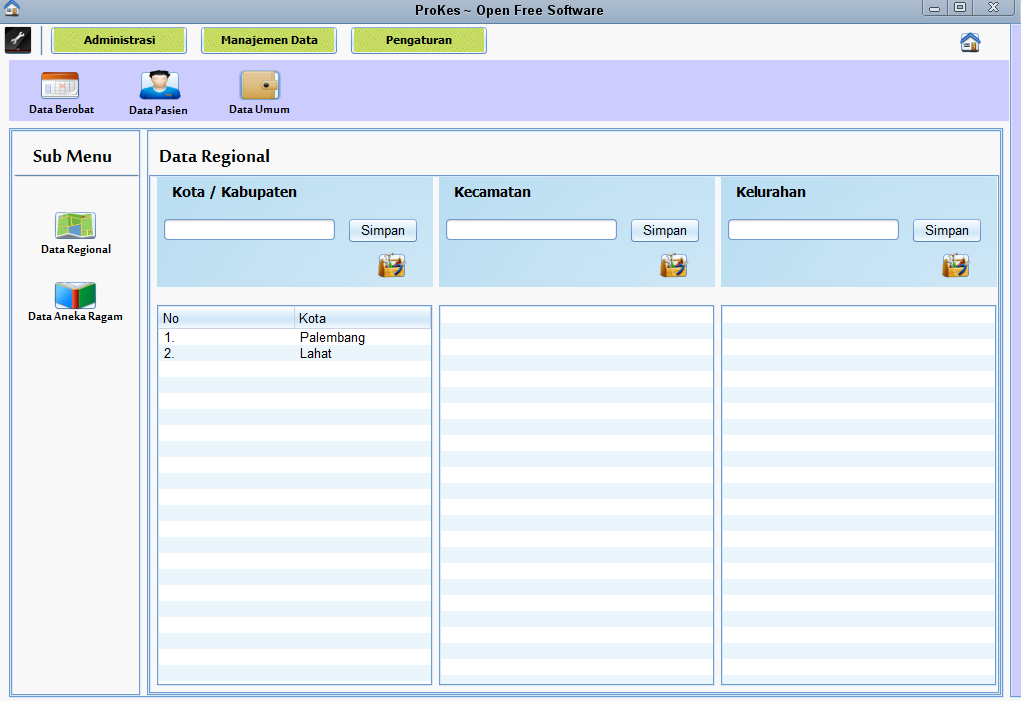


## Figure 9

The general data sub-menu consists of two sub-menus, namely:

1. Regional Data

This sub-menu functions to enter the city/district, sub-district and village data under the area where the health unit is located.

1. Varied Data


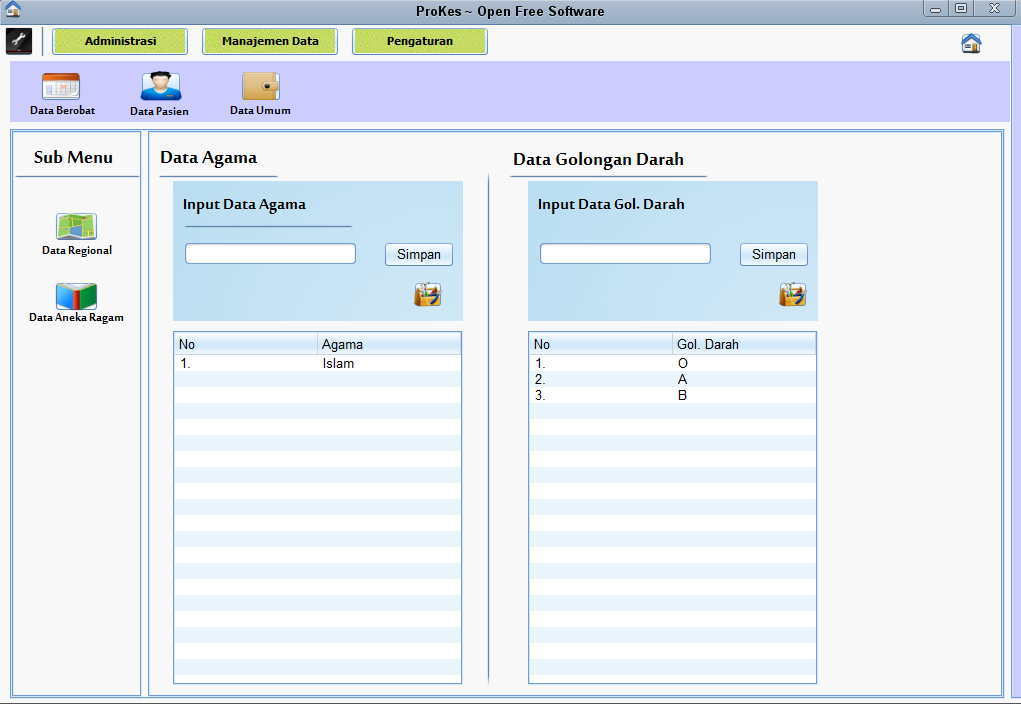


## Figure 10

In this sub-menu, the user can enter religious data as well as blood-type data.

1. **Settings Menu**

This menu consists of three sub-menus, namely:

1. Database

e
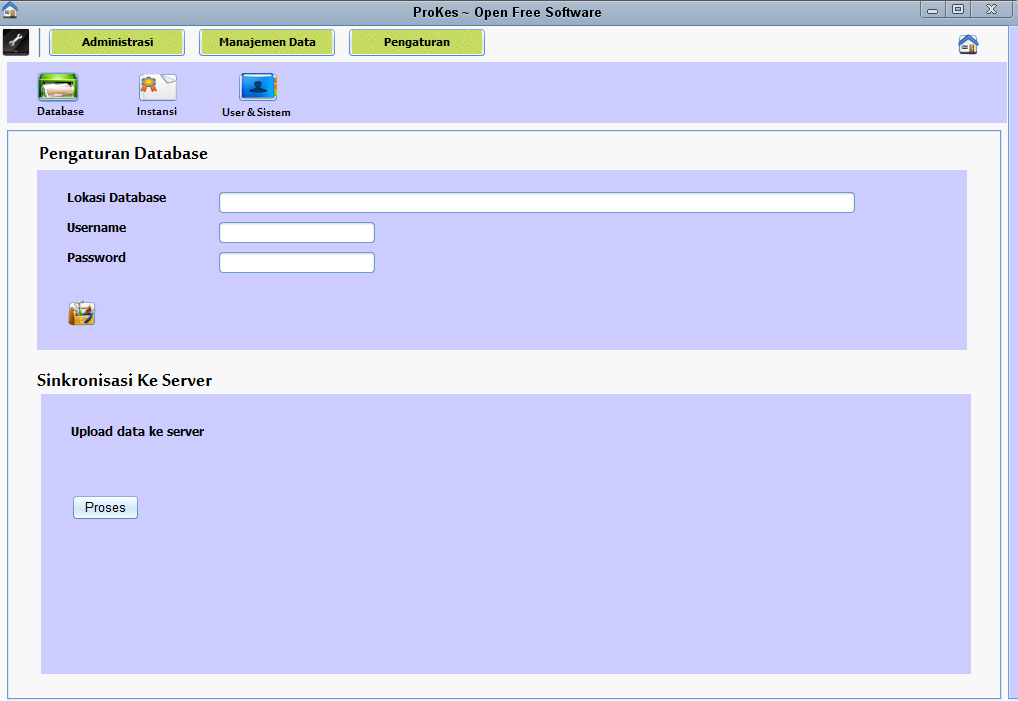


## Figure 11

This sub-menu contains the database address settings that are used to store the application's data. There are also menus for synchronising data and uploading data to a central server.

1. Government Agency


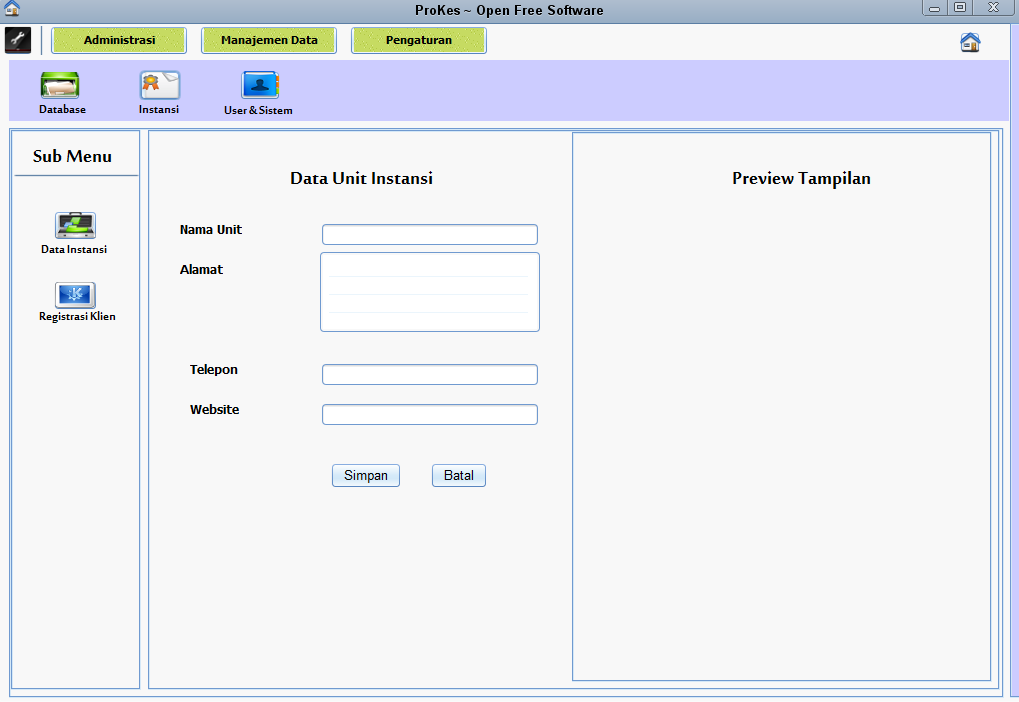


## Figure 12

1. User System


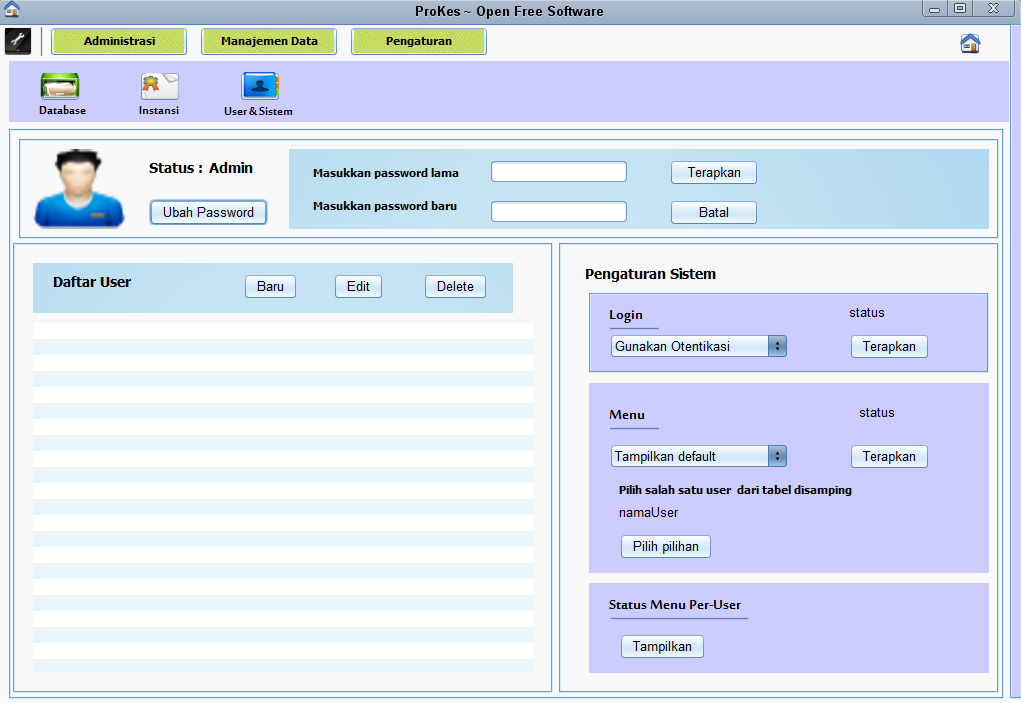


## Figure 13

Contains user data tables that have been registered; this sub-menu is specifically for administration users.

In addition, this sub-menu contains application system settings such as login settings, whether using automatic or username and password authentication, menu settings and also which users of user-type applications can access the menus.

**Head of Department**

The following will show the use of a web-based MRIS for the Head of the district health office, who has access rights to view reports, overall graphs, graphics per district and graphics for each village. To obtain permission to enter the service, the Head of the district health office must log in. First, the login page is shown in Figure 14.


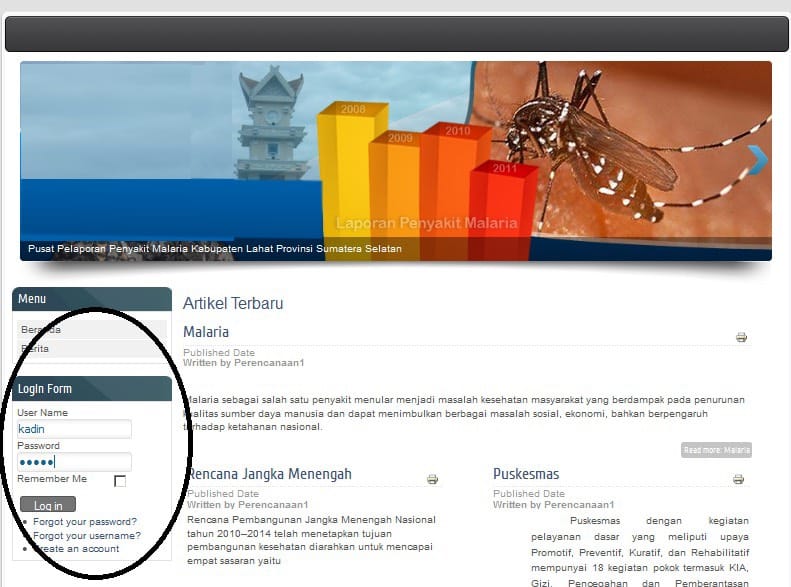


## Figure 14

Enter username and password in the circle above.

If the login has been successful, then a page will appear as in Figure 15.


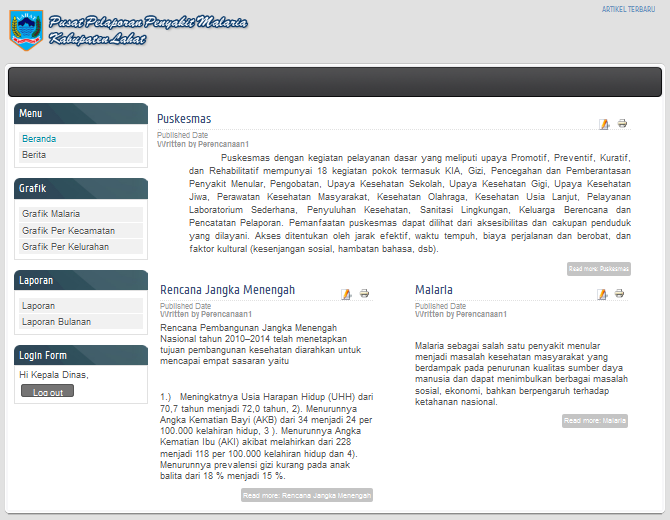


## Figure 15

Figure 16 contains menus that are useful for displaying gateways, news, malaria charts, graphs per district, graphs for each village and monthly reports, as well as overall reports.

1. The homepage and news page are useful for displaying all the latest news that has been posted by the planning department; this page is shown in Figure 16.


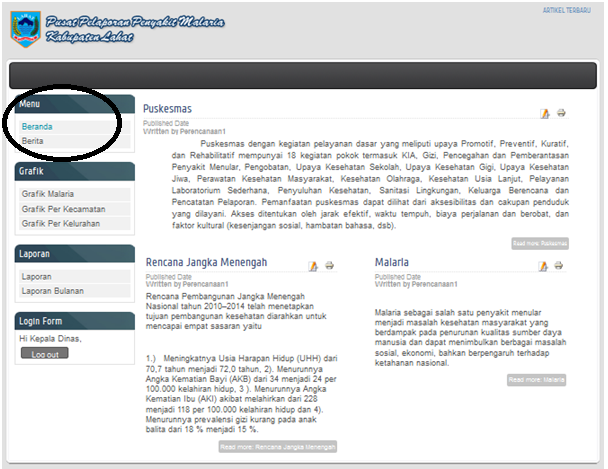


## Figure 16

1. Graph Page of Malaria

This page is used for displaying graphs of malaria as a whole per year, an example of this is shown in Figure 17.


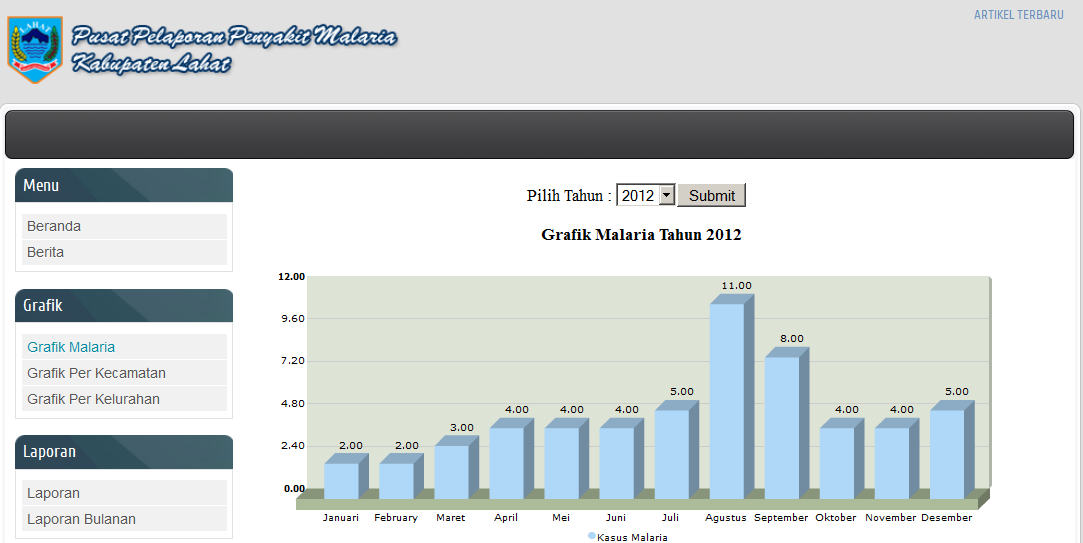


## Figure 17

1. Graph Pages Per District

This page is used for displaying graphs of malaria as a whole per year, an example of this is shown in Figure 18.


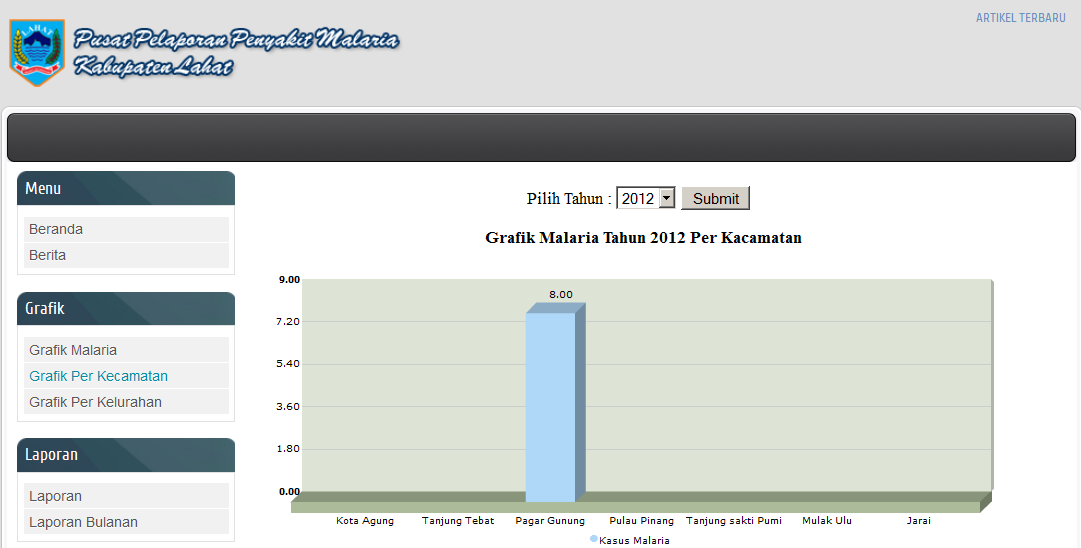


## Figure 18

1. Graph Pages for Each Village

This page is used for displaying graphs of malaria per village as a whole per year, an example of this is shown in Figure 19.


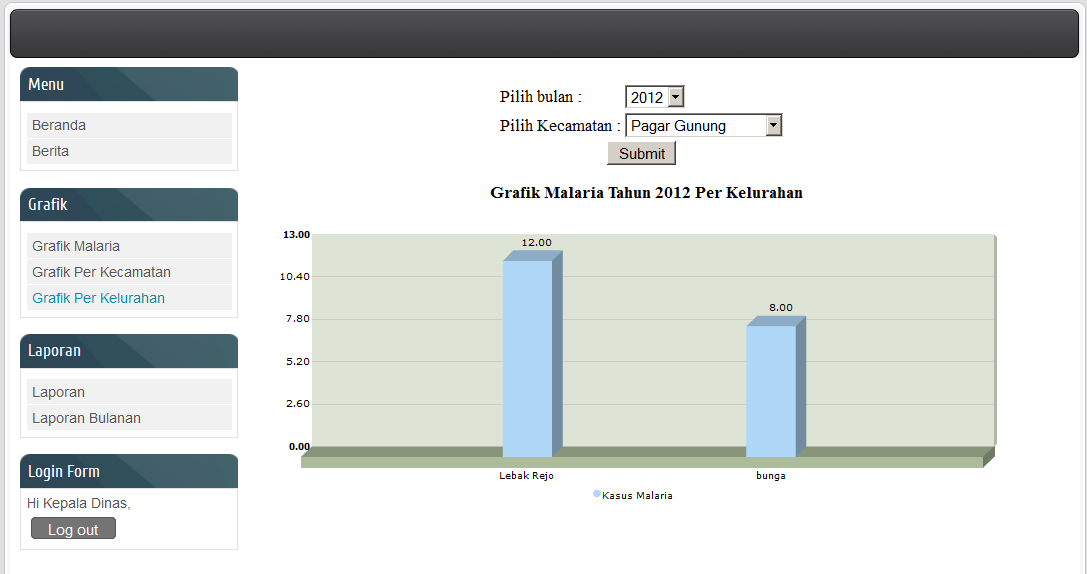


## Figure 19

1. Report Page

This page is used for displaying malaria case reports, an example of which is shown in Figure 20.


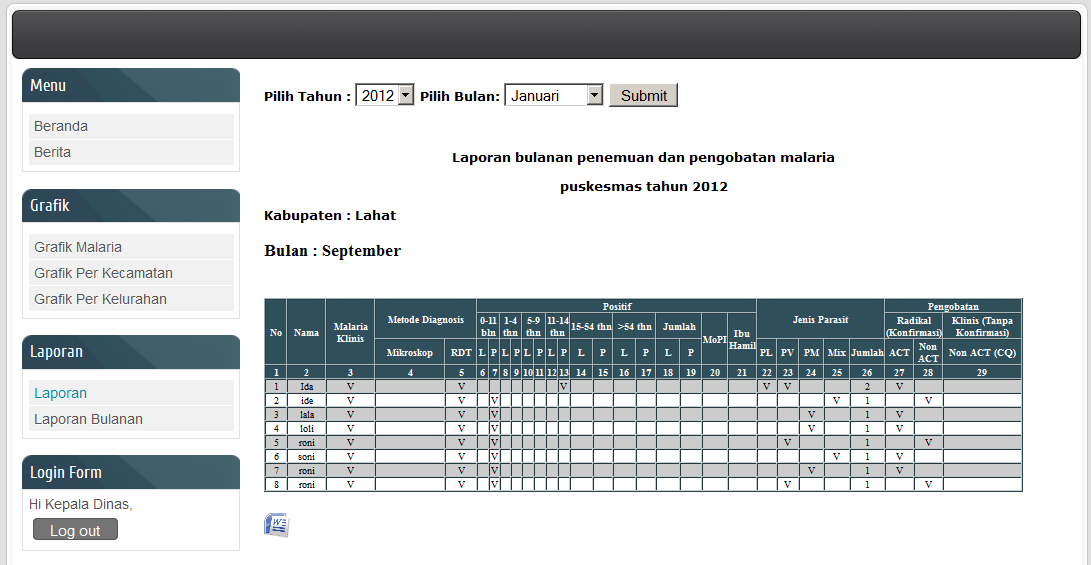


## Figure 20.

1. Monthly Reports Page

Thispage is used for displaying reports of malaria cases regularly in 1 month, as shown in Figure 21.


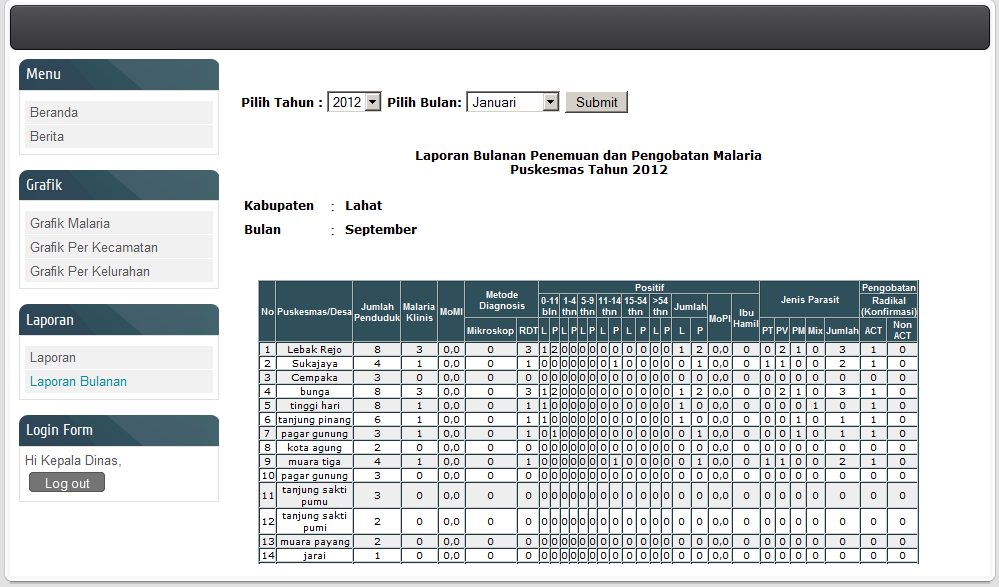


## Figure 21

Thus, the entire display is on the Head's office access page.

**Planning Section**

The following will show the use of a web-based MRIS for the planning section. The planning section has access rights to view reports, overall graphs, graphics per district and graphics for each village and to post news on the web-based MRIS system. To enter access rights to the planning section, one must login first; the login page is shown in Figure 22.


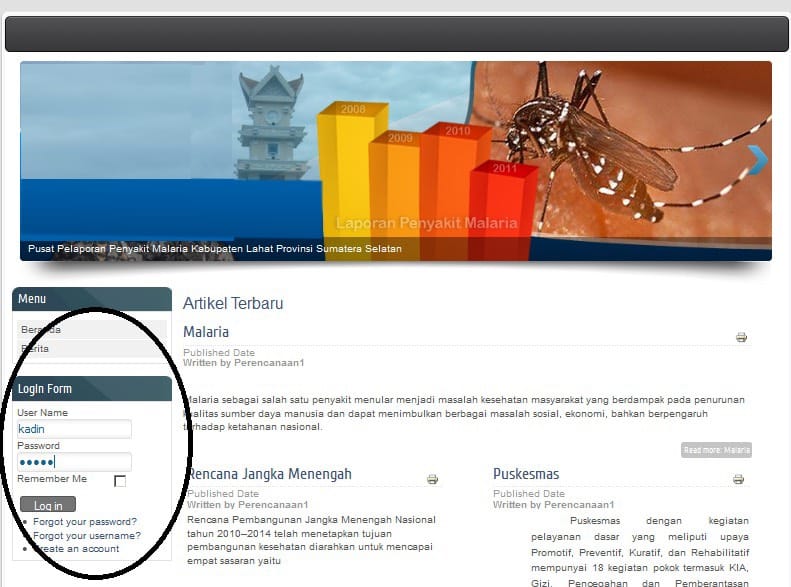


## Figure 22

Enter username and password in the circle above.

If the login has been successful, then a page will appear as in Figure 23.


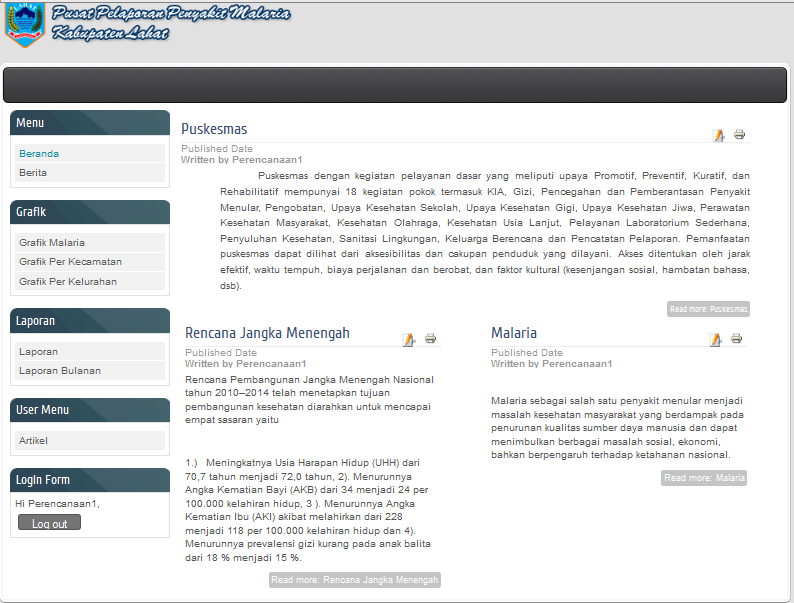


## Figure 23

Figures 24 contains menus that are useful for displaying gateways, news, malaria charts, graphs per district, graphs for each village, overall reports, monthly reports and articles.

1. The homepage and news page are useful for displaying all the latest news that has been posted by the planning section; the page is displayed in Figure 24.


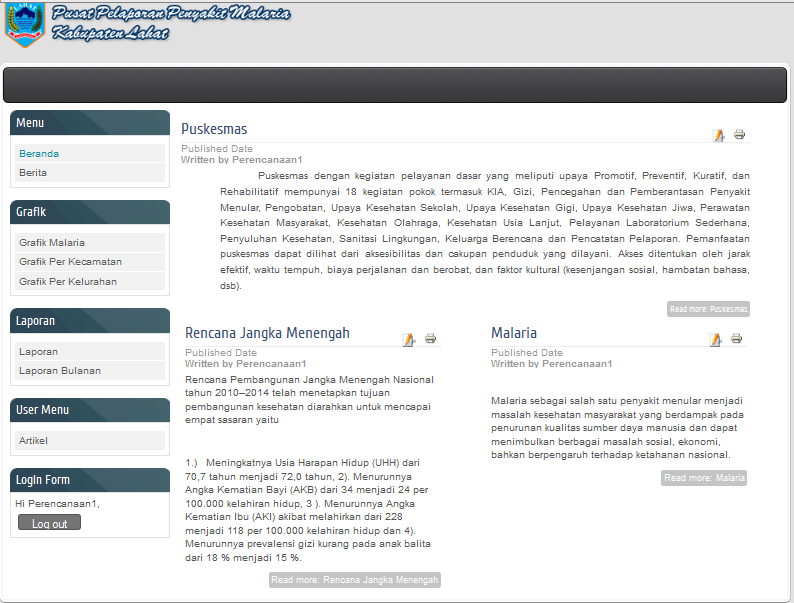


## Figure 24

1. Malaria Graph Page

This page is used for displaying a graph of malaria as a whole per year, an example of this is shown in Figure 25.


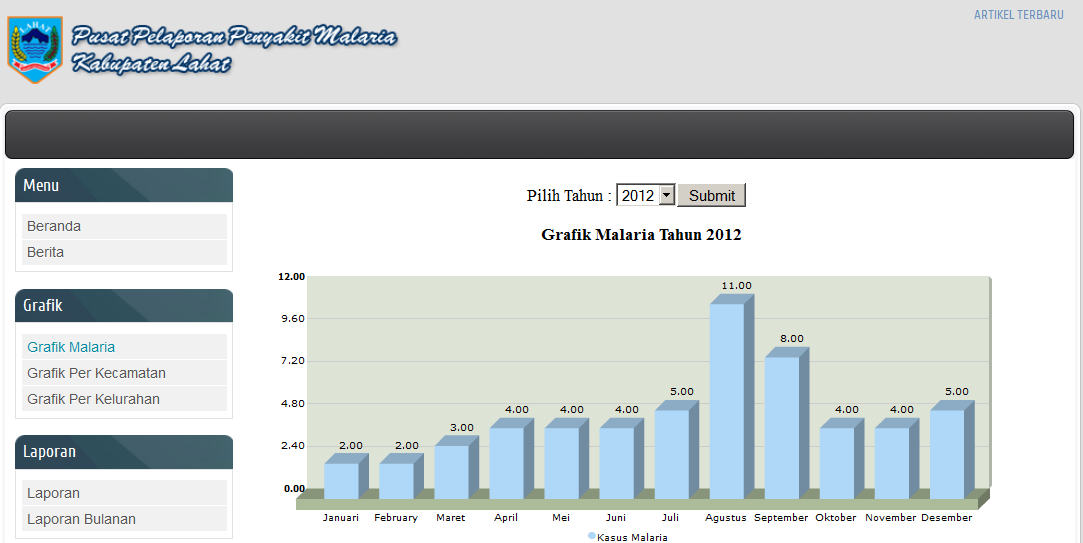


## Figure 25

1. Graph Pages Per District

This page is used for displaying graphs of malaria per district as a whole per year, an example of this is shown in Figure 26.


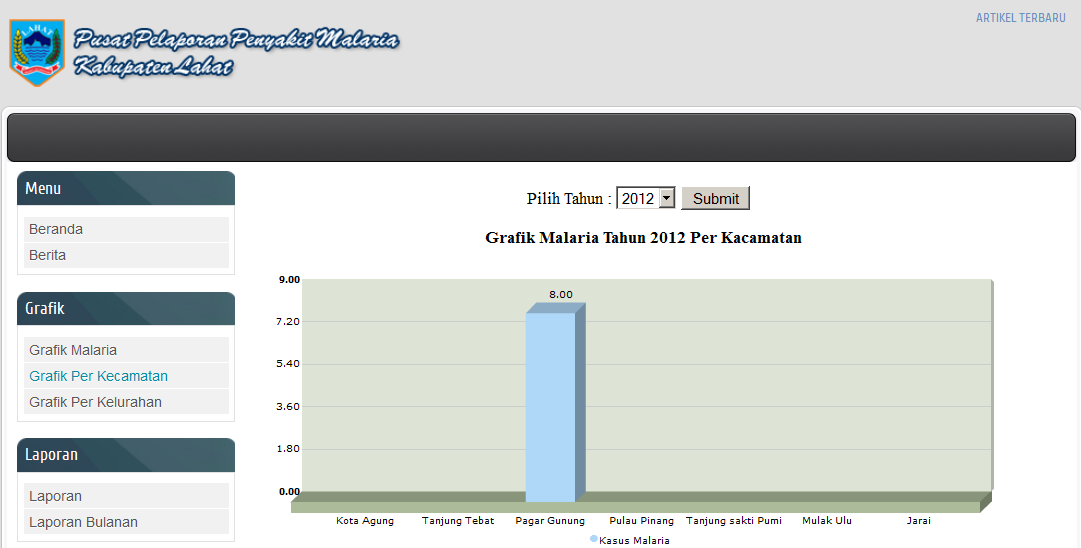


## Figure 26

1. Graph Pages Per Village

This page is used for displaying graphs of malaria per village as a whole per year, an example of this is shown in Figure 27.


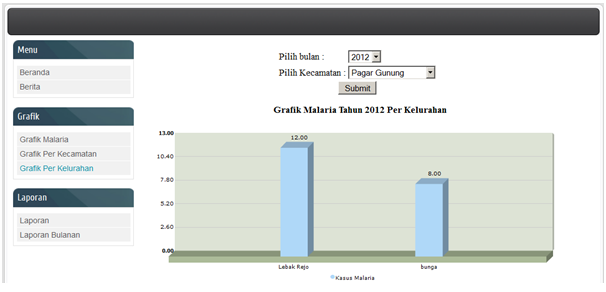


## Figure 27

1. Report Page

This page is used for displaying malaria case reports, an example of this page isshown in Figure 28.


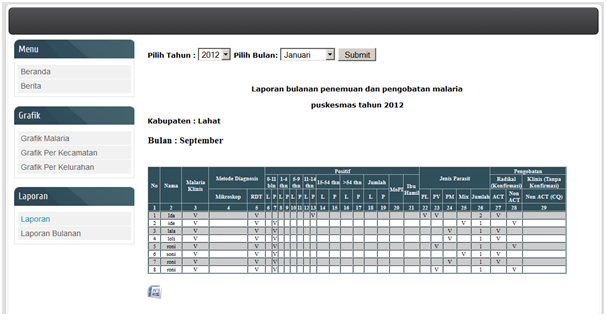


## Figure 28

1. Monthly Report Page

This page is used for displaying reports of malaria cases regularly in 1 month, an example of this is shown in Figure 29.


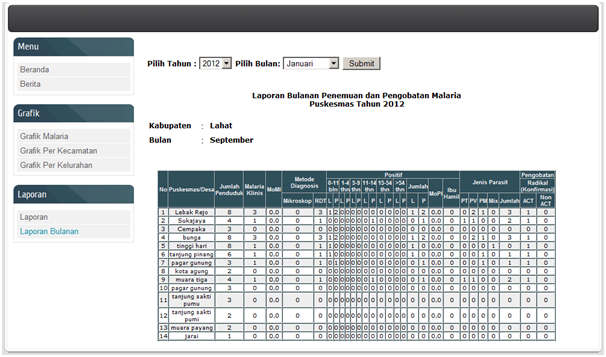


## Figure 29

1. Article Pages

This page is used for adding news/articles to the following SIPP system on the article page, an example of this is shown in Figure 30.


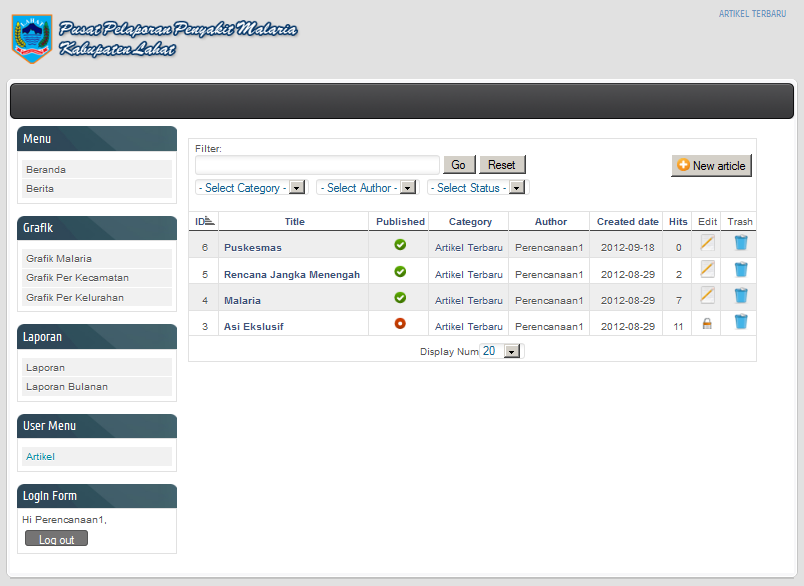


## Figure 30

Thus, the planning section has access to the entire display. Based on input from the informants in the review, a web-based version of the MRIS at the Lahat district health office requires:

1. *Instructions for use: the clarity of each function described must still be given several explanations in detail, especially at the level of application management authority, the order / procedure for entering data into reports, relating the news to the public and whether there is already a filter or editorial board that is responsible. Regarding the security of data shared with several institutions, the form of real reports that exist when printed on paper should be attached. The colouring for some parts must be denser,* *if the monitor is of lower resolution, then the colour will not be true and difficult to interpret*
2. *Basic data whose changes are not dynamic, such as primary health care centre codes and some other data, can still be made by combo box or list box only.*
3. *The tables can be ordered in ascending or descending order so that information will be captured more quickly, including the use of colour classifications for variables or things that pass the usual standard or are less than the reasonable standard. A warning code for important and crucial data obtained from the results of data processing would be useful, this would also serve to monitor the validity of the data and the reliability of the instruments used by data providers.*
4. *Standard health centre report formats in the form of spreadsheets should also be provided, with several formats used by private parties to facilitate the reading of information or to provide them on several possible scales (A4, Quarto, Folio, etc. ).*
5. *The use of open-sources does facilitate development, but the open-source still has to build its security system that is well structured so that the access code is not easily penetrated by malware or the like.*
6. *Sentinel blanks for routine information should, if possible, be filled in with the time the data was taken and the name of the data inputter, with the thought to trace back to this if the data is suspected of having input errors.*
7. *The output can be added to some content related to standard recommendations for information obtained, based on evidence.*
