# Supplementary material for: Potential for a web-based management information system to improve malaria control: An exploratory study in the Lahat District, South Sumatra Province, Indonesia
Source: PLoS One. 2020 Jun 9;15(6):e0229838. doi: 10.1371/journal.pone.0229838 (PMC7282623; doi:10.1371/journal.pone.0229838)
Supplement: S1 Table — (DOCX) [file pone.0229838.s003.docx]

Table 1. **Components of the Prototype Feasibility Test**

| No | **Component** |
| --- | --- |
| A | **Component of the Input Design** |
| 1 | Full input  Facility to validate and verify data entry |
| 2 | User acceptable  Users can easily use input forms including logic and visual graphics |
| 3 | Data back up mechanism  It has a direct entry device as a substitute for source documents in the event of a system locking |
| B | **Components of the Process Design** |
| 1 | System Operating Procedure  Efficient and effective processing procedures |
| 2 | Reliable Software  The software has consistency and reliability in carrying out maximum activities with optimal results |
| 3 | Facilities and Functions  All facilities and functions both logic, mathematical, statistical, visual, automation functions can be active well |
| 4 | Modelling  The software system has a flexible model for the appropriate problem case |
| 5 | Time Accuracy  Conversion of input to output has the efficiency and effectiveness of time that is appropriate both in time running and response time |
| C | **Database Design Components** |
| 1 | Data Back-Up  Data has a secure data back up mechanism |
| 2 | Security System Database |
| 3 | Entities and Attributes  Clear identity, description following the contents, the identity of the data file under the process program |
| 4 | Relational Database  Showing table relations, precise and accurate query responses, primary keys are consistent, fast and correct |
| 5 | Data Flow  Data flow from input to the database is appropriate and actual, and it has a zero error rate. |
| 6 | Database Capacity  Loads a lot of data but the accuracy and speed of access is efficient and effective |
| D | **Components of Full Design** |
| 1 | Supporting Policy  Applied to interests that suit your needs |
| 2 | Recovery System Mechanisms  Able to recover systemic damage in the event of a disaster |
| 3 | Simulation System  Having facilities and operational instructions for the user |
| 4 | Access Control System  Users have access level security system. |
| E | **Components of a Technology Platform Design** |
| 1 | Technology  The latest minimum and maximum technology can accept the system |
| 2 | Configuration  It's easy to get technology configurations for running the system and optimal system configuration procedures |
| 3 | System Capacity  The system has an efficient and effective installation size according to the needs and technological capabilities |
| 4 | Response and Running Time  The time is taken by the system as a whole to perform the functions of the facility, both input, process, output, and control effectively and efficiently. |
|  | Scoring scale :  100  75  50  25  0    Failed Denied Modification Accept |
|  | The system created based on the book Software Engineering (Raymond McLeod) and Strategic Software Engineering which modified |
